# Patient-centered Care through Difficult Intravenous Access Process Improvements

**DOI:** 10.1097/pq9.0000000000000320

**Published:** 2020-08-07

**Authors:** Jamie Harrison, Audra Bradfield, Kelsey Kowalski

**Affiliations:** From the * PICU Nurse Manager, Nemours Children’s Hospital Nemours Children’s Health System; † Continuous Improvement Resource Office, Nemours Children’s Health System; ‡ Enterprise Strategy and Project Management Office, Nemours Children’s Health System

**Fig. 1.**
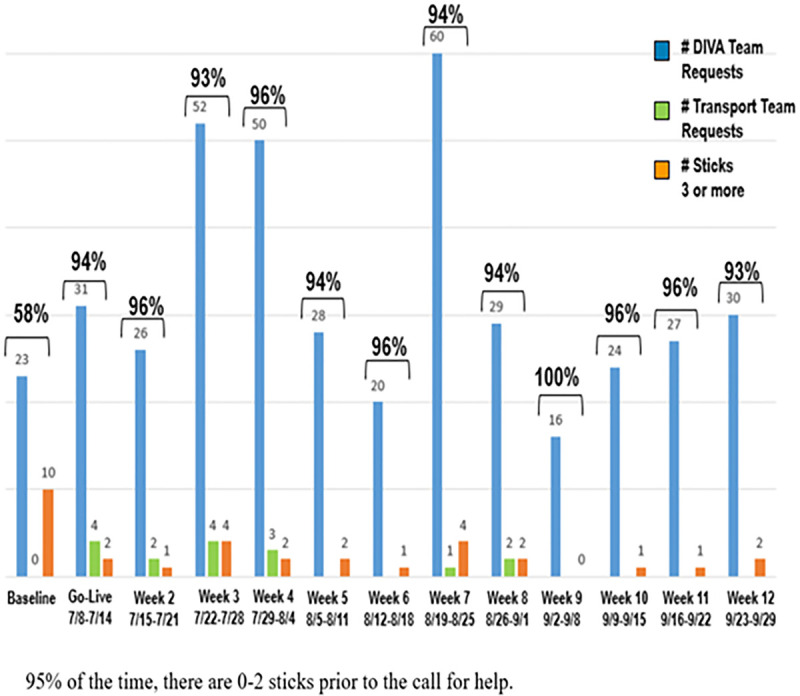
Number of DIVA team requests and number of sticks. Ninety-five percent of time, there are 0–2 sticks before the call for help.

**Fig. 2.**
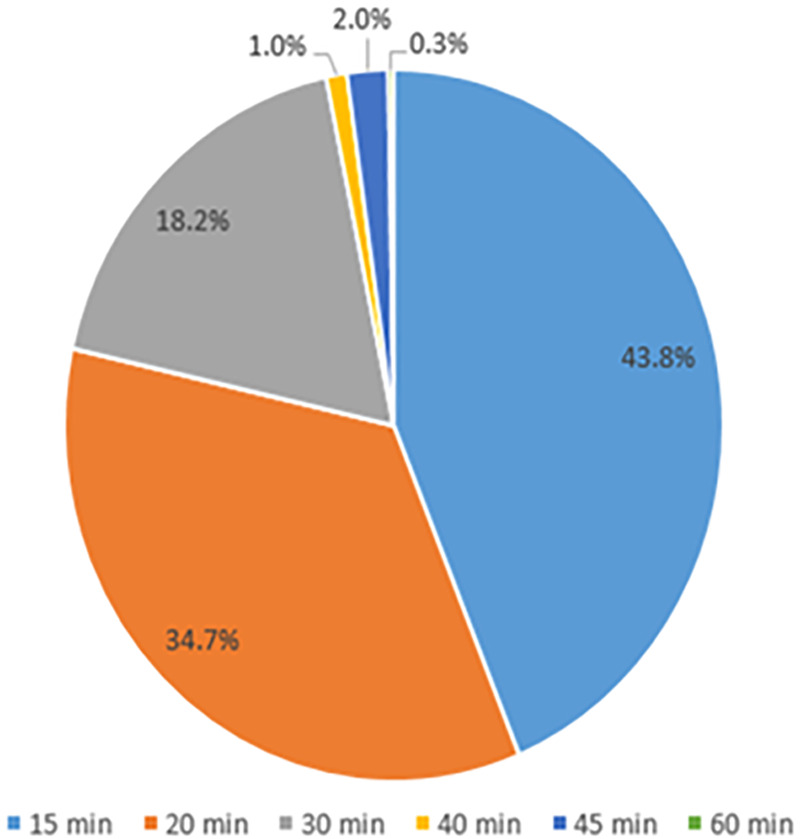
DIVA RN time away from unit.

The PICU team surfaced a problem through their daily huddle that patient care units were attempting multiple “sticks” on patients before seeking help from nurses who were trained in ultrasound guided IV access. After gathering data, this problem was discovered to be frequent and impactful. In just under 7 weeks, IV trained PICU nurses were called approximately 132 times to assist other units with IV insertions. Over 40% of the time, patients had been stuck 3 or more times before help was requested. Some patients had up to 9 attempts. The primary purpose of the project is to prevent undo harm to our patients and utilize our current staff and resources. Goals and targets for the project include: (1) decreasing a number of sticks before escalation. As a baseline, 42% of the time 3 or more sticks occur before an escalation for assistance, and the team aimed for a target of 0–2 sticks 100% of the time before escalation for help. (2) Increase the percentage of patients who receive IV fluid under 60 minutes or less. The baseline for this goal is 72.6% of patients receive IV fluid under 60 minutes or less, with a target of 90% of patients receive IV fluid under 60 minutes or less. Leadership secured support to utilize a Rapid Process Improvement Workshop, an effective lean tool to realize significant improvements quickly. The team engaged the Family Advisory Council for the first-hand patient family feedback and created a new process that included scripting, criteria for decision making, checklists, and escalation pathways. Patient care unit nurses were trained on new process before implementation. Major interventions include: Fifteen days after implementation, 40% of difficult insertion patients receiving 3 or more sticks before the associate asked for help has been reduced to 4%. Ninety-six percent of patients received 0–2 sticks before escalation, and the majority escalated are 0 sticks (Fig. 1). This stability has been maintained at 96% for 50 days. The development of criteria for identification of DIVA patients, escalation pathways, and utilization of ultrasound-guided IV access will decrease the number of attempts at access for DIVA patients. Additionally, the time nurses spent outside of their unit was reduced due to this new process, allowing for less disruption to patient care (Fig. 2).

- Single process flow with one point of contact and clear escalation pathway.
- Establish a difficult intravenous access (DIVA) team using current staff, paged when needed—Pediatric Intensive Care Unit (PICU) nurse, PICU PCT, and Child Life—identified and available for every shift as a Voalte group.
- Utilize an evidence-based DIVA scoring tool on all patients needing IVs before attempt to reduce unnecessary sticks.
- Scripting for bedside nurse and information sheet for nurse to provide to caregiver. Scripting for DIVA nurse developed to discuss ultrasound machine process with patient and caregiver and information sheet.
- DIVA supply kits with checklist and visual indicator to indicate readiness.
- Daily DIVA team huddle to monitor improvement progress via area readiness, and to promptly address Plan Do Check Adjust (PDCA) project needs.

Revised May, 2020: These results have been maintained for over 12 months.

